# A super gene expression system enhances the anti-glioma effects of adenovirus-mediated REIC/Dkk-3 gene therapy

**DOI:** 10.1038/srep33319

**Published:** 2016-09-14

**Authors:** Tetsuo Oka, Kazuhiko Kurozumi, Yosuke Shimazu, Tomotsugu Ichikawa, Joji Ishida, Yoshihiro Otani, Toshihiko Shimizu, Yusuke Tomita, Masakiyo Sakaguchi, Masami Watanabe, Yasutomo Nasu, Hiromi Kumon, Isao Date

**Affiliations:** 1 Department of Neurological Surgery, Okayama University Graduate School of Medicine, Dentistry and Pharmaceutical Sciences, Okayama, Japan; 2 Department of Cell Biology, Okayama University Graduate School of Medicine, Dentistry and Pharmaceutical Sciences, Okayama, Japan; 3 Center for Innovative Clinical Medicine, Okayama University Hospital, Okayama, Japan; 4 Department of Urology, Okayama University Graduate School of Medicine, Dentistry and Pharmaceutical Sciences, Okayama, Japan

## Abstract

Reduced expression in immortalized cells/Dickkopf-3 (REIC/Dkk-3) is a tumor suppressor and therapeutic gene in many human cancers. Recently, an adenovirus REIC vector with the super gene expression system (Ad-SGE-REIC) was developed to increase REIC/Dkk-3 expression and enhance therapeutic effects compared with the conventional adenoviral vector (Ad-CAG-REIC). In this study, we investigated the *in vitro* and *in vivo* effects of Ad-SGE-REIC on malignant glioma. In U87ΔEGFR and GL261 glioma cells, western blotting confirmed that robust upregulation of REIC/Dkk-3 expression occurred in Ad-SGE-REIC-transduced cells, most notably after transduction at a multiplicity of infection of 10. Cytotoxicity assays showed that Ad-SGE-REIC resulted in a time-dependent and significant reduction in the number of malignant glioma cells attaching to the bottom of culture wells. Xenograft and syngeneic mouse intracranial glioma models treated with Ad-SGE-REIC had significantly longer survival than those treated with the control vector Ad-LacZ or with Ad-CAG-REIC. This study demonstrated the anti-glioma effect of Ad-SGE-REIC, which may represent a promising strategy for the treatment of malignant glioma.

Malignant glioma is the most frequent type of primary brain tumor in adults. Glioblastoma, which is highly malignant, is characterized by marked neovascularity, rapid cell proliferation, infiltrative cell migration, and extensive necrosis^1^. The median survival of patients treated aggressively for glioblastoma is approximately 14.6 months^2^. Currently, several new therapeutic agents, including various molecular targeted drugs, are being developed and evaluated in clinical trials.

Reduced expression in immortalized cells/Dickkopf-3 (REIC/Dkk-3) was identified as a gene whose expression is reduced in a variety of human cancer cells^3^,^4^,^5^,^6^. Adenovirus-mediated REIC/Dkk-3 (Ad-REIC) overexpression acts via c-Jun-NH2-kinase (JNK) and c-Jun^5^,^7^ and via endoplasmic reticulum (ER) stress^6^ to induce apoptosis in malignant mesothelioma and in prostate and testicular cancer cells, but not in non-cancer cells. Ad-REIC treatment also inhibits the expression of Id-1, which influences cell cycle progression and has an anti-apoptotic effect^8^.

REIC/Dkk-3 regulates the growth and survival of glioma cells by caspase-dependent and -independent mechanisms via modification of the Wnt signaling pathway^9^. Using western blot analysis, we previously confirmed that REIC/Dkk-3 protein expression was reduced in malignant glioma cell lines^10^. Furthermore, increasing REIC/Dkk-3 expression with an adenovirus vector led to a marked increase in the number of TUNEL-positive cells. The *REIC*/*Dkk-3* gene regulates cell growth through caspase-dependent apoptosis, in particular, via caspase-9. Moreover, increasing REIC/Dkk-3 expression decreases β-catenin expression. These findings suggest that intracellular overexpression of REIC/Dkk-3 plays a distinct role in apoptosis induction and anti-oncogenic activity. However, there are only a few reports on the immunological reaction to secretory or exogenous REIC/Dkk-3 protein^11^,^12^,^13^.

Gene therapy-based approaches often require high levels of gene expression and protein products^14^,^15^,^16^,^17^. We developed a novel adenoviral vector expressing REIC/Dkk-3, based on the cytomegalovirus (CMV) promoter-driven super gene expression system (Ad-SGE-REIC), by inserting the triple translational enhancer sequences of human telomerase reverse transcriptase (hTERT), Simian virus 40 (SV40), and CMV, downstream of the bovine growth hormone polyadenylation (BGH polyA) sequence. This gene expression cassette was named the super gene expression (SGE) system^18^. Because the CMV promoter-SGE system facilitates more potent gene expression, Ad-SGE-REIC is superior to conventional adenoviral systems with respect to REIC protein expression and therapeutic effects in prostate, renal, and cervical cancer and in malignant mesothelioma.

In this study, we compared Ad-SGE-REIC with a conventional Ad-REIC vector and evaluated the anti-glioma effect of Ad-SGE-REIC against malignant glioma. We further tested the effect of the activated immune system in a syngeneic mouse glioma model.

## Results

### Overexpression of REIC/Dkk-3 protein with Ad-SGE-REIC versus Ad-CAG-REIC

**Figure 1:**
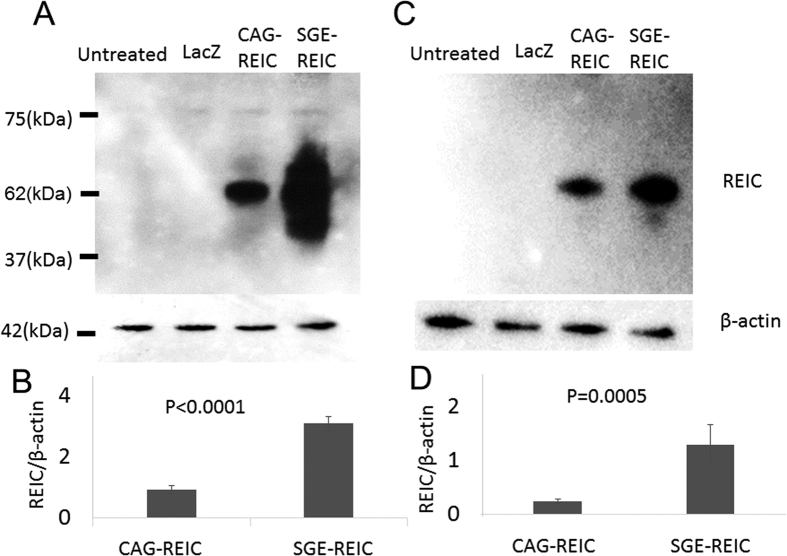
Protein expression of REIC/Dkk-3 in U87ΔEGFR and GL261 glioma cells after treatment with Ad-SGE-REIC or Ad-CAG-REIC. U87ΔEGFR and GL261 glioma cells were infected with Ad-SGE-REIC or Ad-CAG-REIC at an MOI of 10. (**A**) In U87ΔEGFR glioma cells, the increase in expression levels of REIC/Dkk-3 protein was greater after Ad-SGE-REIC treatment than after Ad-CAG-REIC treatment. (**B**) Quantification of the expression ratio (average expression levels: Ad-CAG-REIC; 0.93, Ad-SGE-REIC; 3.1) (n = 4). The protein band density was calculated using ImageJ software. P < 0.001. (**C**) In GL261 glioma cells, the increase in expression levels of REIC/Dkk-3 protein was greater after treatment with Ad-SGE-REIC than with Ad-CAG-REIC. (**D**) Quantification of the expression ratio (average expression levels: Ad-CAG-REIC; 0.25, Ad-SGE-REIC; 1.3) (n = 4). The protein band density was calculated using ImageJ software. P = 0.005. Data are shown as the mean ± SD.

To examine the potential of REIC/Dkk-3 as a tool for targeted gene-based therapy, REIC/Dkk-3 was overexpressed using Ad-SGE-REIC in comparison with Ad-CAG-REIC. An adenoviral vector carrying the LacZ gene with a CAG promoter (Ad-LacZ) was used as the control. These adenoviral vectors were generated using replication-defective adenoviruses of serotype 5. REIC/Dkk-3 protein levels in U87ΔEGFR and GL261 glioma cells were evaluated at 36 h after treatment with Ad-CAG-REIC or Ad-SGE-REIC. Robust upregulation of REIC/Dkk-3 expression was observed in the Ad-SGE-REIC-transduced cells at a multiplicity of infection (MOI) of 10 (Fig. 1).

### Cytotoxic effect of Ad-SGE-REIC compared with Ad-CAG-REIC

**Figure 2:**
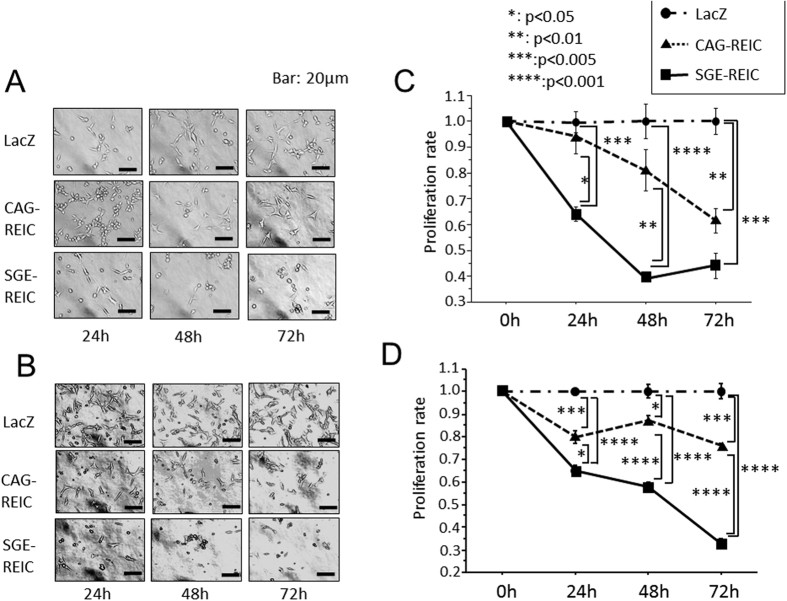
Cytotoxicity after Ad-SGE-REIC treatment in glioma cell lines. U87ΔEGFR (**A**,**C**) and GL261 (**B**,**D**) glioma cells were infected with Ad-SGE-REIC, Ad-CAG-REIC, or Ad-LacZ at an MOI of 10. Cell viability was examined 24, 48, and 72 h after infection. In cytotoxicity assays, the proliferation rate of malignant glioma cells was reduced in a time-dependent manner after treatment with Ad-SGE-REIC and the effect was stronger compared with that of Ad-CAG-REIC (*p < 0.05, **p < 0.01, ***p < 0.005, ****p < 0.001).

Initially, glioma cells were infected with adenovirus, the adenovirus-containing media were aspirated at 3 h after infection, and the cells were then incubated in fresh media. The *in vitro* cytotoxic effect of Ad-REIC on glioma cells was investigated. U87ΔEGFR and GL261 cell lines were incubated with Ad-LacZ, Ad-CAG-REIC, or Ad-SGE-REIC at an MOI of 10 for the indicated times. The proliferation rates of both types of malignant glioma cells were time-dependently and more substantially reduced by Ad-SGE-REIC relative to Ad-CAG-REIC and Ad-LacZ (Fig. 2).

### Cytotoxicity of Ad-SGE-REIC against normal human astrocytes

**Figure 3:**
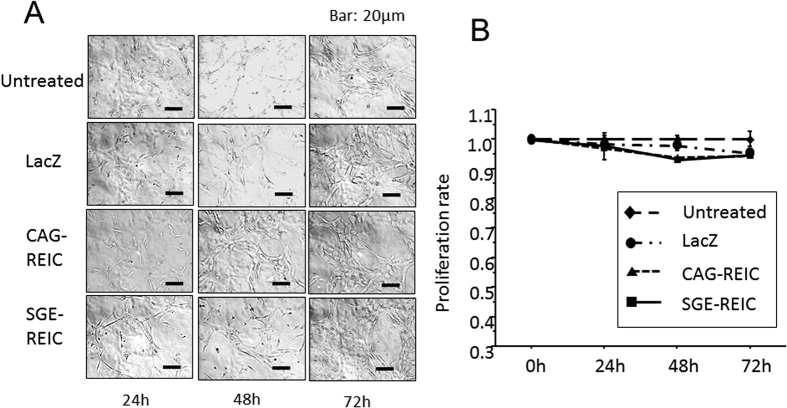
Cytotoxicity after Ad-SGE-REIC treatment in normal human astrocytes (NHA). NHA cells were infected with Ad-SGE-REIC, Ad-CAG-REIC, or Ad-LacZ at a MOI of 10. Cell viability was examined 24, 48, and 72 h after infection. In cytotoxicity assays, the proliferation rates of NHAs were not significantly different after treatment with Ad-SGE-REIC, Ad-CAG-REIC, or Ad-LacZ.

The *in vitro* cytotoxic effect of Ad-REIC on normal human astrocyte (NHA) cells was investigated. Incubation with Ad-LacZ, Ad-CAG-REIC, or Ad-SGE-REIC at an MOI of 10 for the indicated time did not alter the proliferation rate of NHA cells (Fig. 3).

### Caspase expression, ER stress, and β-catenin degradation by REIC/Dkk-3 in malignant glioma cells

**Figure 4:**
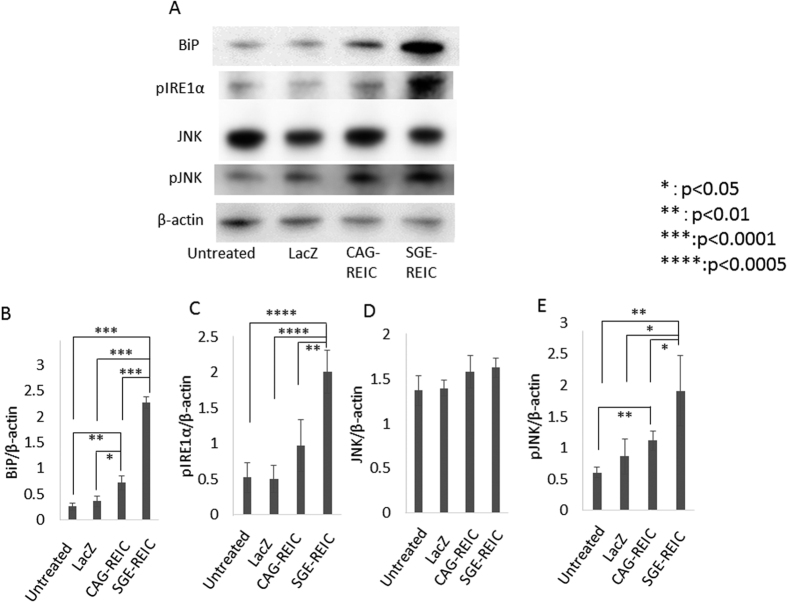
ER stress in U87ΔEGFR glioma cells after treatment with Ad-SGE-REIC. U87ΔEGFR cells were infected with Ad-SGE-REIC, Ad-CAG-REIC, or Ad-LacZ at a MOI of 10. Immunoblot analysis showed that levels of BiP, phosphorylated IRE1α, SAPK/JNK, and phosphorylated SAPK/JNK were increased in the U87ΔEGFR cell line following treatment with Ad-SGE-REIC. (**B**) Quantification of the expression ratio of BiP (average expression levels: Ad-CAG-REIC; 0.72, Ad-SGE-REIC; 2.27) (n = 4). (**C**) Quantification of the expression ratio of pIRE1α (average expression levels: Ad-CAG-REIC; 0.96, Ad-SGE-REIC; 2.01) (n = 4). (**D**) Quantification of the expression ratio of SAPK/JNK (average expression levels: Ad-CAG-REIC; 1.58, Ad-SGE-REIC; 1.62) (n = 4). (**E**) Quantification of the expression ratio of pSAPK/JNK (average expression levels: Ad-CAG-REIC; 1.11, Ad-SGE-REIC; 1.90) (n = 4). Protein band density was calculated using ImageJ software. Data are shown as the mean ± SD. *p < 0.05, **p < 0.01, ***p < 0.0001, ****p < 0.0005.

U87ΔEGFR glioma cells were treated with Ad-LacZ, Ad-CAG-REIC, or Ad-SGE-REIC at an MOI of 10. At 36 h after infection, glioma cells were harvested. Western blot analysis revealed increased expressions of ER stress marker molecules Bip, phosphorylated IRE1α, and phosphorylated SAPK/JNK in Ad-SGE-REIC-infected cells compared with those in Ad-CAG-REIC- and Ad-LacZ-infected cells (Fig. 4).

The Wnt signaling pathway additionally regulates cell survival by inhibition of proteasome-dependent proteolysis of β-catenin. Therefore, we evaluated the impact of Ad-LacZ, Ad-CAG-REIC, and Ad-SGE-REIC treatment on β-catenin expression in malignant glioma cells. β-catenin protein levels were more potently reduced by Ad-SGE-REIC treatment than by Ad-CAG-REIC treatment.

**Figure 5:**
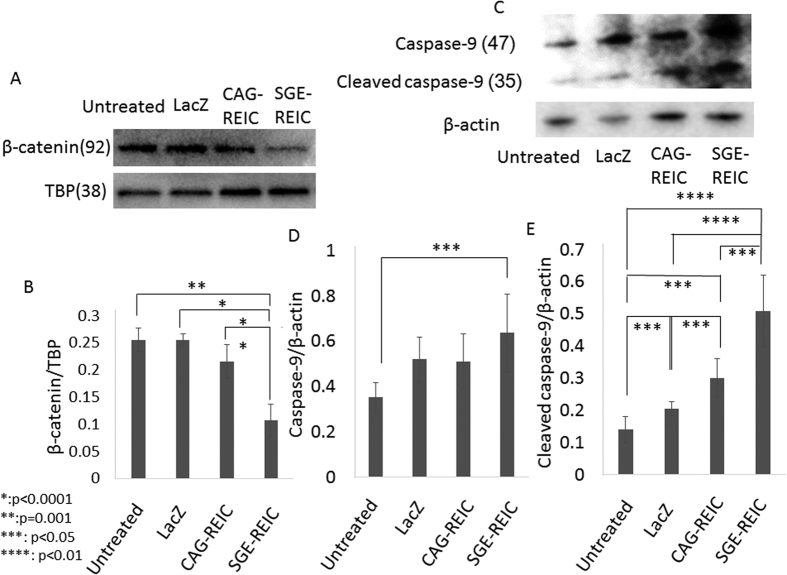
Expression of β-catenin in the nucleus of U87ΔEGFR glioma cells and caspase-9 expression in U87ΔEGFR glioma cells after Ad-SGE-REIC treatment. (**A**) U87ΔEGFR cells were infected with Ad-SGE-REIC, Ad-CAG-REIC, or Ad-LacZ at an MOI of 10. A reduction in β-catenin expression occurred in parallel with increased expression of REIC/Dkk-3 (n = 4). (**B**) Quantification of the expression ratio of β-catenin (average expression levels: Ad-CAG-REIC; 0.22, Ad-SGE-REIC; 0.11) (n = 4). (**C**) Cleaved caspase-9 expression increased following treatment with Ad-SGE-REIC compared with Ad-CAG-REIC or Ad-LacZ. (**D**) Quantification of the expression ratio of caspase-9 (average expression levels: Ad-CAG-REIC; 0.51, Ad-SGE-REIC; 0.63) (n = 4). (**E**) Quantification of the expression ratio of cleaved caspase-9 (average expression levels: Ad-CAG-REIC; 0.30, Ad-SGE-REIC; 0.50) (n = 4). Protein band density was calculated using ImageJ software. Data are shown as the mean ± SD. *p < 0.0001, **p = 0.001, ***p < 0.05, ****p < 0.01.

Moreover, the activity of caspase-9 was evaluated in U87ΔEGFR cells. The cleaved form of caspase-9 expression was also increased in cells treated with Ad-SGE-REIC compared with those treated with Ad-CAG-REIC or Ad-LacZ (Fig. 5).

### Therapeutic efficacy of Ad-SGE-REIC in xenograft mouse models and a syngeneic model

**Figure 6:**
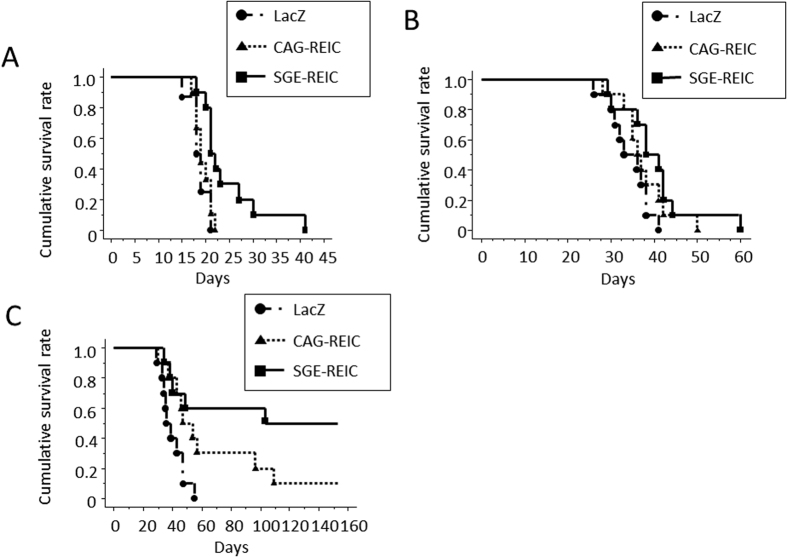
Kaplan-Meier survival curves of the U87ΔEGFR and GL261 mouse glioma models and of the GL261 mouse syngeneic models treated with Ad-SGE-REIC or Ad-CAG-REIC. (**A**) At 7 days after U87ΔEGFR cell implantation to BALB/c mice, mice were treated with Ad-SGE-REIC, Ad-CAG-REIC, or Ad-LacZ (3.6 × 10^7 ^pfu) by direct intratumoral injection. The survival time of mice treated with Ad-SGE-REIC was significantly longer than that of those treated with Ad-LacZ or Ad-CAG-REIC (median survival = 22, 18, and 19 days, respectively; P = 0.0038 and P = 0.0107) (n = 10 each group). (**B**) At 7 days after GL261 cell implantation to BALB/c mice, mice were treated with Ad-SGE-REIC, Ad-CAG-REIC, or Ad-LacZ (3.6 × 10^7 ^pfu) by direct intratumoral injection. The survival time of mice treated with Ad-SGE-REIC was significantly longer than that of those treated with Ad-LacZ (median survival = 41 and 33 days; P = 0.0257) (n = 10 each group). (**C**) At 7 days after GL261 cell implantation to C57BL/6N mice, mice were treated with Ad-SGE-REIC, Ad-CAG-REIC, or Ad-LacZ (3.6 × 10^7 ^pfu) by direct intratumoral injection. The survival time of mice treated with Ad-CAG-REIC was significantly longer than that of those treated with Ad-LacZ (median survival = 47 and 36 days, respectively; P = 0.024). The survival time of mice treated with Ad-SGE-REIC was significantly longer than that of those treated with Ad-LacZ (median survival = 103 and 36 days, respectively; P = 0.004) (n = 10 each group).

The anti-tumor effect of Ad-CAG-REIC and Ad-SGE-REIC was tested in mice bearing intracerebral glioma (U87ΔEGFR or GL261) and a syngeneic model (GL261). In all experiments, mice were injected with 3.6 × 10^7^ plaque-forming units (pfu) of adenovirus. Kaplan-Meier curves were used to analyze the survival time of the U87ΔEGFR mouse glioma model after treatment with Ad-LacZ, Ad-CAG-REIC, or Ad-SGE-REIC. The median survival time was longer in mice treated with Ad-SGE-REIC than with Ad-LacZ (22 and 18 days, respectively; P = 0.0038; Fig. 6A). Median survival was also longer in mice treated with Ad-SGE-REIC than with Ad-CAG-REIC (22 and 19 days, respectively; P = 0.0107; Fig. 6A). In the GL261 mouse glioma model, the median survival time was also significantly longer in mice treated with Ad-SGE-REIC than with Ad-LacZ (41 and 33 days, respectively; P = 0.0257; Fig. 6B).

In the GL261 syngeneic model, the median survival time of mice treated with Ad-CAG-REIC was significantly longer than that of those treated with Ad-LacZ (47 and 36 days, respectively; P = 0.024; Fig. 6C). The median survival time of mice treated with Ad-SGE-REIC was also significantly longer than that of those treated with Ad-LacZ (103 and 36 days, respectively; P = 0.004; Fig. 6C).

### Infection with Ad-REIC induces lymphocyte and dendritic cell infiltration into glioma

**Figure 7:**
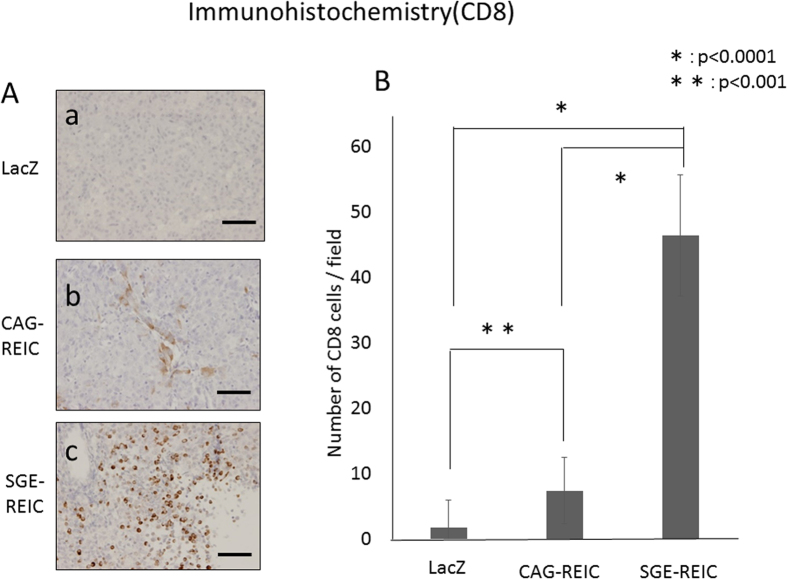
Histological analysis of glioma treated with Ad-REIC. CD8-positive killer T lymphocyte infiltration into gliomas treated with Ad-SGE-REIC and with Ad-CAG-REIC was detected by monoclonal antibody staining. A significant increase in CD8-positive cells was detected in gliomas treated with Ad-SGE-REIC compared with Ad-CAG-REIC (*P < 0.0001).

**Figure 8:**
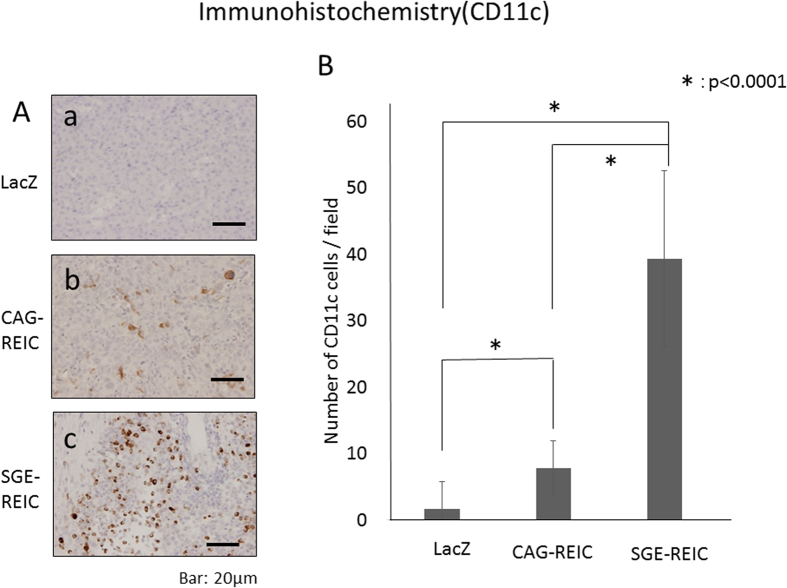
Histological analysis of glioma treated with Ad-REIC. CD11c-positive dendritic cell infiltration in gliomas treated with Ad-SGE-REIC and with Ad-CAG-REIC was detected by monoclonal antibody staining. A significant increase in CD11c-positive cells was observed in gliomas treated with Ad-SGE-REIC compared with Ad-CAG-REIC (*P < 0.0001).

Immunological reactions to Ad-REIC were investigated by histological evaluation of GL261 gliomas at 21 days after viral infection. Few CD8- and CD11c-positive cells had infiltrated the tumor specimens treated with Ad-LacZ (Figs 7 and 8). In stark contrast, obvious infiltration of both CD8- and CD11c-positive cells was detected in tumors treated with Ad-SGE-REIC or Ad-CAG-REIC (Figs 7 and 8). Infiltration of CD8- and CD11c-positive cells was significantly greater in tumors treated with Ad-SGE-REIC relative to those treated with Ad-CAG-REIC (P < 0.0001).

## Discussion

### Summary of results

This study focused on the anti-glioma activity of Ad-SGE-REIC, a novel adenoviral vector that produces higher protein expression and a superior therapeutic effect compared with the conventional system (Ad-CAG-REIC). REIC/Dkk-3 expression was upregulated in Ad-SGE-REIC-transduced glioma cells, and the most prominent effect was obtained after transduction at 10 MOI. In cytotoxicity assays, Ad-SGE-REIC time-dependently reduced the number of viable malignant glioma cells. In xenograft and syngeneic intracranial glioma models, Ad-SGE-REIC was associated with significantly longer survival than Ad-LacZ or Ad-CAG-REIC. Infiltration of CD8- and CD11c-positive cells was significantly greater in syngeneic gliomas treated with Ad-SGE-REIC than in those treated with Ad-CAG-REIC.

### Effects of Ad-REIC on glioma

Expression levels of REIC/Dkk-3 mRNA and protein are downregulated in malignant glioma cell lines^10^. Mizobuchi *et al*. reported that overexpression of REIC/Dkk-3 with a plasmid vector induced apoptosis in malignant glioma cells^9^. Similarly, we previously found that enhancing REIC/Dkk-3 expression with an adenoviral vector led to a marked increase in the number of TUNEL-positive cells. Our data indicated that levels of the activated form of caspase-9 were significantly higher in glioma cells treated with Ad-SGE-REIC than in those treated with Ad-CAG-REIC and control.

Moreover, the expressions of Bip, phosphorylated IRE1α, and phosphorylated SAPK/JNK were increased in Ad-SGE-REIC-infected cells compared with Ad-CAG-REIC- and Ad-LacZ-infected cells. This result indicated that ER stress was strongly evoked by Ad-SGE-REIC. ER stress was also found to be evoked by enhanced REIC/Dkk-3 expression in malignant mesothelioma and in prostate and testicular cancer cells^6^,^19^. Additionally, expression levels of β-catenin, a key element of the Wnt signaling pathway, declined in parallel with the increase in REIC/Dkk-3 expression. Wnt signaling inhibits the release of cytochrome C and the subsequent activation of caspase-9 induced by apoptotic stimuli^20^.

### Ad-SGE-REIC

Watanabe *et al*. found that insertion of the triple translational enhancer sequences of hTERT, SV40, and CMV downstream of the BGH polyA sequence yielded the most potent gene expression^18^. The hTERT promoter/enhancer is well-characterized and has been frequently used for cancer-specific gene expression^21^,^22^,^23^,^24^. Several studies have demonstrated increased gene expression by insertion of the SV40 enhancer downstream of polyA sequences^15^,^16^,^17^. The CMV enhancer is used in the CMV early enhancer/chicken β-actin promoter (CAG promoter), which is known to improve gene expression in various cell types and tissues^16^. Because this novel gene expression system using triple enhancers significantly increases the expression of the gene(s) of interest in comparison with conventional systems using the strong CMV promoter, we termed this novel gene expression cassette, the SGE system.

### Efficacy of Ad-SGE-REIC

In various types of human cancer cell, the induction of apoptosis is significantly increased by transduction of Ad-SGE-REIC compared with conventional Ad-REIC vectors. Furthermore, the inhibitory effects of Ad-REIC treatment on tumor growth have been analyzed in xenograft models. In both mouse renal cell carcinoma and human prostate cancer models, strong suppression of tumor growth was observed in the Ad-SGE-REIC-treated groups relative to the other treatment groups^18^. Thus, the novel SGE system significantly augments the anti-tumor effects of Ad-REIC in mouse xenograft models, and the Ad-SGE-REIC vector was superior to the conventional Ad-CMV-REIC and Ad-CAG-REIC vectors in terms of the efficacy of *in vivo* intratumoral gene therapy. The present findings demonstrated that in xenograft models the survival time of mice treated with Ad-SGE-REIC was significantly longer than that of those treated with Ad-LacZ or Ad-CAG-REIC. Furthermore, in a syngeneic model, the survival time of mice treated with Ad-SGE-REIC was vastly longer than that of those treated with conventional Ad-REIC.

### Anti-tumor effect of Ad-SGE-REIC in the syngeneic model

In the GL261 syngeneic mouse glioma model, mice treated with Ad-CAG-REIC survived significantly longer than those treated with Ad-LacZ. Infiltration of CD8- and CD11c-positive cells was significantly greater in tumors treated with Ad-CAG-REIC than in those treated with Ad-LacZ. In another study, intratumoral administration of REIC/Dkk-3 protein also significantly suppressed tumor growth, which was linked to accumulation of CD8- and CD11c-postiive cells (killer T marker and dendritic cells, respectively), and enhanced the anti-cancer cytolytic activity of splenocytes^11^. Furthermore, the survival time of mice treated with Ad-SGE-REIC was significantly longer than that of those treated with Ad-LacZ. Both CD8- and CD11c-positive cells displayed significantly greater infiltration into tumors treated with Ad-SGE-REIC than into those treated with Ad-CAG-REIC. Therefore, the *in vivo* anti-tumor effect of REIC/Dkk-3 protein largely depends on the induction of enhanced systemic anti-cancer immunity.

### Future direction

Ad-REIC is being developed for evaluation in clinical trials. At the time of publication, a first-in-human, phase I/IIa clinical trial of *in situ* Ad-REIC gene therapy for prostate cancer was done at Okayama University Hospital^25^,^26^. In addition, a phase I clinical trial of Ad-SGE-REIC for malignant mesothelioma was initiated in September 2015. According to the findings of these trials, a clinical trial of Ad-SGE-REIC for the treatment of glioma will be planned. Moreover, we showed that integrin antagonist cilengitide augmented the therapeutic effect of Ad-REIC gene therapy for malignant glioma^10^. Several preclinical studies have shown that cilengitide has an enhanced antitumor effect when administered in combinatorial therapeutic regimens^27^,^28^,^29^,^30^,^31^,^32^,^33^. Furthermore, combination therapy of Ad-REIC with chemotherapy, molecular targeted therapy, and immunotherapy should also be evaluated.

In conclusion, we demonstrated the anti-glioma effect of the Ad-SGE-REIC. Our results indicated that Ad-SGE-REIC has potential as a strategy for the treatment of malignant glioma.

## Materials and Methods

### Cell lines

The glioma cell lines U87ΔEGFR and GL261 were seeded on tissue culture dishes (BD Falcon, Franklin Lakes, NJ, USA) and cultured in Dulbecco’s modified Eagle’s medium supplemented with 10% fetal bovine serum, 100 U penicillin, and 0.1 mg/ml of streptomycin. GL261 cells were provided by Dr. A. Natsume, Nagoya University (Nagoya, Japan). NHA cells were purchased from Takara Bio Inc. (Shiga, Japan).

### Adenovirus vector carrying SGE-REIC/Dkk-3

For Ad-REIC under the control of the CAG promoter, the full-length human REIC/Dkk-3 gene was inserted into the cosmid vector pAxCAwt and then transferred into an adenoviral vector using the COS-TPC method (Takara Bio). The SGE system was made by inserting the triple translational enhancer sequences of human telomerase reverse transcriptase (hTERT), Simian virus 40 (SV40), and cytomegalovirus (CMV) downstream of the BGH polyA sequence. An adenoviral vector carrying the LacZ gene with a CAG promoter (Ad-LacZ) was used as the control. These adenoviral vectors were generated using replication-defective adenoviruses of serotype 5^18^.

### Cytotoxicity assay

Cells were cultured in flat-bottomed six-well dishes at a concentration of 4.0 × 10^5 ^cells/well. The cells were infected with Ad-SGE-REIC, Ad-CAG-REIC, or Ad-LacZ at an MOI of 10.

At 24, 48 and 72 h later, Cell viability was examined. The number of cells attached to the bottom of each culture well was determined in three different wells using a Z2 Coulter Counter (Beckman Coulter, Brea, CA, USA).

### Western blot analysis

After cell culture in flat-bottomed six-well dishes, the media were aspirated, the dishes were washed twice in phosphate-buffered saline, and the cells were lysed in 1% sodium dodecyl sulfate. The lysates were sonicated for analyzing whole-cell proteins. Nuclear proteins were isolated using an NE-PER Nuclear and Cytoplasmic Extraction Kit (Thermo Scientific, Waltham, MA, USA), according to the manufacturer’s instructions. Extracted protein samples were separated by gel electrophoresis and transferred onto polyvinylidene difluoride membranes. After blocking in 5% skim milk, the membranes were incubated overnight with primary antibodies at 4 °C. The membranes were washed with Tris-buffered saline-Tween 20 (TBST), incubated with secondary antibodies at room temperature for 1 h, and rinsed with TBST. Signals were visualized using the ECL Prime Western Blotting Detection System (GE Healthcare Life Sciences, Buckinghamshire, UK). The primary antibodies, mouse anti-human REIC/DKK-3 (provided by the Department of Urology, Okayama University, Okayama, Japan), rabbit anti-human Bip, mouse anti-human β-catenin, rabbit anti-human caspase-9, rabbit anti-human SAPK/JNK, mouse anti-human phosphorylated SAPK/JNK, TATA-binding protein (TBP) (Cell Signaling Technology, Danvers, MA, USA), and rabbit anti-human phosphorylated IRE1α (Novus Biochemicals, Littleton, CO, USA) were diluted 1:1000 in TBST, and β-actin antibody (Sigma, St Louis, MO, USA) was diluted 1:5000 in TBST. The secondary antibody horseradish peroxidase-conjugated anti-mouse or anti-rabbit IgG (Cell Signaling Technology) was diluted 1:6000 (REIC/Dkk-3, caspase-9, and β-actin) or 1:2000 (β-catenin, BiP, pIRE1α, SAPK/JNK, pSAPK/JNK, and TATA-binding protein) in TBST.

### Ethics and animal use statement

This study was conducted in strict accordance to the recommendations in the Guide for the Care and Use of Laboratory Animals in Japan. Animals were housed at 25 °C with 12-h light/dark cycles and free access to water and standard rodent chow in the Department of Animal Resources of Okayama University. All procedures and animal protocols were approved by the Committee on the Ethics of Animal Experimentation at Okayama University (Permit No. OKU-2014264). All surgery was performed under general anesthesia with ketamine/pentobarbital, and all efforts were made to minimize animal suffering.

### *In vivo* experiments

Before implantation, 85 to 90% confluent U87ΔEGFR cells or GL261 cells were trypsinized and centrifuged at 100 *g* for 5 min; the cell pellet was resuspended in phosphate-buffered saline, and the cell concentration was adjusted to 1.0 × 10^5 ^cells/μl. For the xenograft models, U87ΔEGFR or GL261 cells (2 μl) were injected into 6-week-old female BALB/c nude mice (CLEA Japan Inc., Tokyo, Japan). For the syngeneic model, GL261 cells (2 μl) were injected into 6-week-old female C57BL/6N mice. The mice were anesthetized and placed in stereotactic frames (Narishige, Tokyo, Japan) with their skulls exposed. Tumor cells were injected with a Hamilton syringe (Hamilton, Reno, NV, USA) into the right frontal lobe (3 mm lateral to the midline, 1 mm posterior to the coronal suture, 3 mm depth from the dura), and the syringe was withdrawn slowly after 5 min to prevent reflux. The skulls were then cleaned, and the incision was sutured. At 7 days after tumor inoculation, all mice bearing brain tumors were reanesthetized and stereotactically injected with Ad-SGE-REIC, Ad-CAG-REIC, or Ad-LacZ at the tumor inoculation site using the same coordinates.

### Histological procedures

For the detection of CD8- or CD11c-positive cell infiltration into gliomas after Ad-REIC treatment, GL261 glioma cells were implanted, then 3.6 × 10^7 ^pfu of Ad-SGE-REIC, Ad-CAG-REIC, or Ad-LacZ was injected intratumorally 7 days after tumor inoculation. Mice were sacrificed, and their excised brains were embedded in paraffin at 28 days after tumor inoculation. Immunohistochemical staining was performed after samples were deparaffinized in xylene and rehydrated in decreasing concentrations of ethanol. Sections with a thickness of 4 μm were incubated in 0.3% H_2_O_2_ (30 min) and then autoclaved for 15 min at 121 °C in 10 mM sodium citrate buffer, pH 6.0. Immunohistochemical staining for CD8 was performed with mouse monoclonal CD8 antibody (1:50 dilution, no. 550281, BD Pharmingen, San Diego, CA, USA). The Dako Cytomation Envision+ System-HRP Kit was then applied according to the manufacturer’s protocol (Dako Cytomation, Carpentaria, CA, USA). After washing in PBS, the sections were counterstained with hematoxylin. Immunohistochemical staining for CD11c was performed with mouse monoclonal anti-CD11c antibody (no. 550375, BD Pharmingen) using the same method.

### Statistical analyses

Data on protein expression obtained by western blotting were analyzed using Student’s t-test. The proliferation rates obtained from cytotoxicity assays were analyzed using one-way analysis of variance (ANOVA) followed by Tukey’s post hoc test. Kaplan-Meier survival curves were compared using the log-rank test. The number of CD8- and CD11c-positive cells/field was analyzed using one-way ANOVA followed by Tukey’s post hoc test. Statistical analyses were performed using SPSS statistical software (version 20; SPSS, Inc., Chicago, IL, USA). P-values < 0.05 were considered statistically significant.

## Additional Information

**How to cite this article**: Oka, T. *et al*. A super gene expression system enhances the anti-glioma effects of adenovirus-mediated REIC/Dkk-3 gene therapy. *Sci. Rep*. **6**, 33319; doi: 10.1038/srep33319 (2016).
